# Glucocorticoid receptor gene polymorphism and juvenile idiopathic arthritis

**DOI:** 10.1186/1546-0096-9-2

**Published:** 2011-01-13

**Authors:** Mikhail M Kostik, Alexandra A Klyushina, Mikhail V Moskalenko, Larisa A Scheplyagina, Valentina I Larionova

**Affiliations:** 1 Hospital Pediatric Department, Saint-Petersburg State Pediatric Medical Academy, Saint-Petersburg, Russian Federation; 2 Laboratory of Molecular Biology and Genetics, Federal Heart, Blood and Endocrinology Center, Saint-Petersburg, Russian Federation; 3 Russian Institute of Hematology and Transfusiology, Saint-Petersburg, Russian Federation; 4 Laboratory of Ecology, Federal Scientific Clinical Center of Pediatric Hematology, Oncology and Immunology, Moscow, Russian Federation; 5 Laboratory of Molecular Diagnostics, Saint-Petersburg State Pediatric Medical Academy, Saint-Petersburg, Russian Federation

## Abstract

**Background:**

The glucocorticoid receptor gene (NR3C1) has been suggested as a candidate gene affecting juvenile idiopathic arthritis (JIA) course and prognosis. The purpose of this study is to investigate the glucocorticoid receptor gene *Bcl*I polymorphism (rs41423247) in JIA patients, the gene's role in susceptibility to juvenile idiopathic arthritis, and its associations with JIA activity, course and bone mineralization.

**Methods:**

One hundred twenty-two Caucasian children with JIA and 143 healthy ethnically matched controls were studied. We checked markers of clinical and laboratory activity: morning stiffness, Ritchie Articular Index (RAI), swollen joint count (SJC), tender joint count (TJC), physician's visual analog scale (VAS), hemoglobin level (Hb), leukocyte count (L), platelet count (Pl), Westergren erythrocyte sedimentation rate (ESR), C-reactive protein (CRP), albumin, DAS and DAS28. Bone mineralization was measured by dual-energy X-ray absorptiometry (DXA) of lumbar spine L1-L4. Assessments of bone metabolism included osteocalcin, C-terminal telopeptide (CTT), parathyroid hormone (PTH), total and ionized calcium, inorganic phosphate and total alkaline phosphatase (TAP). *Bcl*I polymorphism was genotyped by polymerase chain reaction restriction fragment length polymorphism.

**Results:**

No association was observed between glucocorticoid receptor gene polymorphism and the presence or absence of JIA. In girls with JIA, the presence of the G allele was associated with an unfavorable arthritis course, a younger age of onset of arthritis (p = 0.0017), and higher inflammatory activity. The higher inflammatory activity was demonstrated by the following: increased time of morning stiffness (p = 0.02), VAS (p = 0.014), RAI (p = 0.048), DAS (p = 0.035), DAS28 (p = 0.05), Pl (p = 0.003), L (p = 0.046), CRP (p = 0.01). In addition, these patients had bone metabolism disturbances as follows: decreased BA (p = 0.0001), BMC (p = 0.00007), BMD (0.005) and Z score (p = 0.002); and higher levels of osteocalcin (p = 0.03), CTT (p = 0.036), TAP activity (p = 0.01) and ionized calcium (p = 0.017). In boys with JIA, no significant differences were observed related to the polymorphic alleles or genotypes.

**Conclusions:**

We suggest that G allele and the GG genotype of the glucocorticoid receptor gene *Bcl*I polymorphism contribute to an unfavorable course and low bone mineral density in girls with JIA.

## Background

Juvenile idiopathic arthritis (JIA) is a heterogeneous group of chronic inflammatory diseases with different degrees of joint involvement, functional disability and decreased quality of life. JIA is classified into different types, which vary in number and type of affected joints, associated organ involvement, outcomes of joint damage, and response to treatment [1]. Hypothalamic-pituitary-adrenal axis disorders are associated with human autoimmune diseases and have also been demonstrated in experimental animal models of arthritis [2]. Endogenous glucocorticoids have native anti-inflammatory, cytotoxic, and immunosuppressive effects [3]. Disturbances in glucocorticoid secretion or signal transduction into cells through glucocorticoid receptor can result in immune system alterations and excessive inflammation with tissue damage. The balance between pro-inflammatory molecules and endogenous or exogenous anti-inflammatory molecules influences the degree of inflammation, response to therapy, disease course, prognosis, and outcome in inflammatory diseases. Resistance and sensitivity to endogenous glucocorticoids has been associated with immune reaction disorders such as systemic lupus erythematosus, rheumatoid arthritis, inflammatory bowel diseases, glucocorticoid resistant asthma, and multiple sclerosis [2-5].

Glucocorticoids produce anti-inflammatory effects by binding to intracellular glucocorticoid receptor-α (GR-α). This complex is translocated to the nucleus where it interacts with glucocorticoid-responsive elements of different genes. Thereby, glucocorticoids inhibit production of proinflammatory cytokines (such as IL-1, IL-2, IL-3, IL-6, IL-8, TNF-α) and stimulate production of anti-inflammatory cytokines (such as IL-4, IL-10, IL-13 and transforming growth factor-β) [5]. There is a negative feedback loop that may lead to secretion of proinflammatory cytokines (such as TNF-α, IL-1, IL-6, and IL-8), thereby enhancing glucocorticoid receptor expression in the cytoplasm [6]. Besides GR-α, there is another isoform of this gene, GR-β which arises by alternative splicing. GR-β is functionally inactive and cannot bind glucocorticoids, but has a negative effect on GR-α activity. Hyperexpression of GR-β has been noted in some types of autoimmune diseases and may be a cause of intrinsic glucocorticoid resistance.

The therapeutic response to glucocorticoids can depend on the functional condition of the glucocorticoid receptor (GR). Side effects of glucocorticoids, such as Cushing syndrome, bone mineral loss, decreased height velocity, ulcerogenesis, and secondary immunоdeficiency also are associated with the functional condition of the GR [7]. It is likely that both the desirable anti-inflammatory effects and the undesirable side effects of glucocorticoids are associated not only with polymorphisms of the GR gene, but also with complicated interactions with many other genes crucial to inflammation, immune system function, and bone and drug metabolism. GR is present in cells of the synovium (synoviocytes, fibroblasts, lymphocytes, and macrophages) of patients with inflammatory joint disease [8], exerting local effects in joints.

We hypothesize that that different alleles of the glucocorticoid receptor gene influence JIA course and prognosis This hypothesis was suggested by findings that many rheumatoid arthritis patients respond well to exogenous glucocorticoids, but some fail to do so [9,10].

Among the several known functionally relevant alleles of the GR, the alleles comprising the GR *Bcl*I polymorphism (rs 41423247) may be important in JIA. First discovered as a restriction fragment length polymorphism after digestion of GR DNA with *Bcl*I, these alleles are determined by a single nucleotide variation - cytosine (most common) or guanine - at position 647 in intron 2. The allele containing cytosine is referred to as allele C, and the allele containing guanine as allele G, resulting in three possible genotypes at this position: homozygous CC, heterozygous GC and homozygous GG [11]. This polymorphism has been associated with hypertension, higher body mass index, and abdominal obesity [12-15]. In the present study we have investigated the pattern of *Bcl*I polymorphism of the GR gene in JIA patients, and the relationships of the genotypes to JIA course and arthritis activity.

## Methods

### Patients and controls

Approval was obtained from the Saint-Petersburg State Pediatric Medical Academy committee on the ethics of research on human beings. Blood samples were obtained after informed written consent. All specimens for the labs were collected at the same time as the DNA specimens. One hundred and twenty two children with JIA were enrolled in the study. Each child was a patient of the Saint-Petersburg State Pediatric Medical Academy rheumatology clinic. We enrolled all patients who fulfilled the EULAR JIA criteria and agreed to take part in our study. Age of study subjects ranged from 1.5 years to 17 years, with a mean age of 11.3 years ± 4.36 years. There were 43 boys (35.2%) and 79 girls (64.8%). All patients were of Caucasian race. Only one child per family was enrolled. The mean age of disease onset was 7.81 ± 4.66 years. The average duration of the disease at the time of inclusion in our study was 3.57 years ± 3.49 years. All patients were divided into 3 groups according to arthritis type: oligoarticular 68 (55.7%), polyarticular 41 (33.6%) and systemic 13 (10.7%). Extended oligoarticular patients were included in polyarticular group. Thirty patients (24.6%) were treated with corticosteroids. DNA samples were obtained from 143 control subjects without a personal history of autoimmune diseases according to records of general pediatrics (DNA bank of laboratory of molecular diagnostics of Saint-Petersburg State Pediatric Medical Academy).

For assessment of JIA activity, clinical markers and special indexes were used. The clinical markers included: morning stiffness (in minutes), Ritchie Articular Index (RAI), swollen joints count (SJC), tender joints count (TJC), and the physician's visual analog scale (VAS) measured in millimeters on a 100 mm line. Other markers included: hemoglobin level (Hb), leukocyte count (L), platelet count (Pl), Westergren erythrocyte sedimentation rate (ESR), C-reactive protein (CRP), and albumin. DAS and DAS28 indexes were measured in all patients.

Bone mineralization was measured by dual-energy X-ray absorptiometry (DXA) of lumbar spine at L1-L4 (Hologic QDR 4500C densitometer with reference pediatric database). Densitometry parameters, such as bone area (BA, cm^2^), bone mineral content (BMC, grams) and bone mineral density (BMD, measured in grams/cm^2 ^and in Z score, SD) were all evaluated. Low bone mineral density for chronological age was defined by Z score < 2 SD, according to the recommendation of the International Society for Clinical Densitometry, 2007.

For assessment of bone metabolism the following measures were used: osteocalcin (bone gla-protein, a marker of osteosynthesis), C-terminal telopeptides (CTT, products of collagen I type degradation and a marker of bone resorption), and parathyroid hormone (PTH) by immunochemiluminiscent assay. Also levels of total and ionized calcium (Ca++), inorganic phosphate and total alkaline phosphatase (TAP) were determined.

### Genetic analysis

Detection of GR gene (NR3C1 - nuclear receptor subfamily 3, group C, member 1) *Bcl*I-polymorphism (rs 41423247) was accomplished by the following previously published method [16]: Polymerase chain reactions (20 μl final volume) contained 1 μg of genomic DNA, 250 pmol of each primer, 10мМ Tris-HCI pH 8.4, 0.5mM MgCI2, 50mM KCl, 0.2mМ dNTPs and 1U Taq polymerase with the following conditions: initial denaturation at 94°C for 5 min; 40 cycles of denaturation at 94°C for 30 s, annealing at 59°C for 30 s, and extension at 72°C for 45 s; followed by a final extension at 72°C for 7 min. The primers used were (5'to 3') for intron B *BclI *SNP: forward primer 5' AAATTGAAGCTTAACAATTTTGGC 3' and reverse primer 5' GCAGTGAACAGTGTACCAGACC 3'. The PCR product had a length 206 bp. To detect the presence of the *Bcl*I polymorphism, this PCR product was digested by *Ksp *22I endonuclease ("Sybenzyme", Russia - specificity identical to *Bcl*I) followed by separation in agarose gel electrophoresis. Individuals with the CC genotype had 2 fragments of 90 and 116 bp due to the presence of the *Bcl*I site. Individuals with the GG genotype had an uncut fragment of 206 bp. Those who were heterozygous (GC genotype) had fragments of 90, 116, and 206 bp. Allele G, also known as the 4.5 kb allele or the long allele, is a minor allele [13].

### Statistical analysis

Associations between the GR polymorphisms and JIA markers and course were analyzed with the SPSS for Windows, release 11. We utilized t-test, chi-square test, and correlation analysis. Because we had a restricted number of JIA boys with GG genotypes for statistical analysis, all boys were divided in two groups: carriers of the G allele (GG and GC genotypes) and carriers of CC genotype (no G allele). P-values < 0.05 were considered to indicate a significant difference.

## Results

The distribution of *Bcl*I genotypes and alleles in JIA patients and controls, separately in girls, boys, and total group, is shown in Table 1. There were no differences between results found in JIA cases and in healthy controls, with no deviation from Hardy-Weinberg equilibrium. In regression analysis the role of the G allele in risk for JIA was not significant (p = 0.43). The frequency of the G allele was 32.3% in oligo JIA, 41.5% in poly JIA, and 42.3% in systemic JIA. Genotypes distribution and sex differences in JIA subtypes shown in Table 2.

**Table 1:** The distribution of *Bcl*I genotypes and alleles in JIA children and controls.

Girls	CC	CG	GG	p	Boys	CC	CG	GG	p	Total	CC	CG	GG	p
JIA				0.48	JIA				0.13	JIA				0.6
n = 79	30	34	15		n = 43	20	21	2		n = 122	50	55	17	
%	(38.0)	(43.0)	(19.0)		%	(46.5)	(48.9)	(4.6)		%	(41.0)	(45.1)	(13.9)	
Controls					Controls					Controls				
n = 70	25	36	9		n = 73	25	36	12		n = 143	50	72	21	
%	(35.7)	(51.4)	(12.9)		%	(34.2)	(49.3)	(16.5)		%	(35.0)	(50.3)	(14.7)	

Alleles	C	G	p			C	G	p			C	G	p	

JIA	94	64	0.82		JIA	61	25	0.09		JIA	155	89	0.48	
Controls	86	54			Controls	86	60			0.09	Controls	172	114	

**Table 2:** Distribution of *BclI *genotypes and alleles in JIA subtypes and controls.

Subtypes	Oligoartricular		Polyarticular		Systemic		Controls	
Gender	Boys	Girls	Boys	Girls	Boys	Girls	Boys	Girls
	47.0%	53.0%	22.0%	78.0%	15.4%	84.6%	49.0%	51.0%
Genotypes								
CC	16	16	4	9	0	5	25	25
%	50.0%	44.4%	44.4%	28.1%	0.0%	45.4%	34.2%	35.7%
CG	14	14	5	17	2	3	36	36
%	43.8%	38.9%	55.6%	53.1%	100.0%	27.3%	49.3%	51.4%
GG	2	6	0	6	0	3	12	9
%	6.2%	16.7%	0.0%	18.8%	0.0%	27.3%	16.5%	12.9%
*p (vs Controls)*	0.19	0.47	0.41	0.63	0.37	0.26		

We analyzed whether differences in arthritis markers between boys and girls depended on polymorphic genotypes. There was a positive correlation between the presence of the G allele and inflammatory markers, such as ESR (r = 0.21), CRP (r = 0.21), and platelet count (r = 0.19). There was a negative correlation with albumin level (r = -0.17). In regression analysis, the presence of the G allele had a significant influence on younger age of onset of JIA (p = 0.02), morning stiffness (p = 0.04), SJC (p = 0.03), RAI (p = 0.05), DAS (p = 0.02), DAS 28 (p = 0.03), platelet count (p = 0.01) and albumin level (p = 0.05). For other parameters of JIA inflammatory activity, such as TJC, VAS, ESR, CRP, Hb and leukocyte count, the presence of the G allele was insignificant. There also was no significant influence on parameters of bone mineral density - Z-score (p = 0.15) and presence of low bone mineral density for chronological age (p = 0.15). Girls with JIA who have the G allele had an increase in some markers of inflammation and severity of arthritis, and had disease onset at a younger age (Tables 3, 4).

**Table 3:** Arthritis activity markers in JIA girls with polymorphic GR genotypes.

Parameter	CC	GC	GG	**p**_**1**_	**p**_**2**_	**p**_**3**_
Onset age, years	9.07 ± 4.9	6.9 ± 4.8	5.38 ± 3.1	**0.04**	0.09	**0.0017**

Morning stiffness, min	66.2 ± 75.0	103.4 ± 85.8	129.0 ± 98.5	**0.03**	0.19	**0.02**

SJC	5.53 ± 6.74	6.03 ± 7.2	11.8 ± 14.1	0.39	0.07	0.06

TJC	7.03 ± 8.0	7.8 ± 6.72	14.7 ± 20.3	0.37	0.11	0.09

VAS, mm	36.3 ± 20.8	39.0 ± 22.4	55.0 ± 27.5	**0.02**	**0.03**	**0.014**

RAI	12.37 ± 14.3	15.3 ± 19.1	23.9 ± 23.4	0.24	0.11	**0.048**

DAS	2.97 ± 1.7	5.3 ± 1.7	4.3 ± 2.4	0.25	0.07	**0.035**

DAS 28	3.44 ± 1.5	3.77 ± 1.4	4.3 ± 1.7	0.19	0.14	**0.05**

Hemoglobin, g/l	123.0 ± 14.5	119.8 ± 14.3	118.5 ± 15.6	0.18	0.39	0.18

Leukocytes, ×10^9^/l	7.61 ± 2.26	8.78 ± 3.2	8.66 ± 2.5	**0.046**	0.45	0.09

Platelets, ×10^9^/l	232.3 ± 60.2	283.5 ± 83.8	286.3 ± 95.3	**0.003**	0.46	**0.03**

ESR, mm/h	14.5 ± 19.3	14.3 ± 14.7	15.6 ± 15.3	0.48	0.39	0.42

CRP, mg/l	4.0 ± 9.6	19.3 ± 37.5	18.4 ± 30.8	**0.01**	0.46	**0.048**

Albumin, %	55.5 ± 6.9	51.5 ± 8.5	50.9 ± 8.5	**0.02**	0.41	**0.04**

р_1 _- comparison between carriers СС and GC genotypes

р_2 _- comparison between carriers GC and GG genotypes

р_3 _- comparison between carriers СС and GG genotypes

**Table 4:** Arthritis activity markers in JIA boys with polymorphic GR genotypes.

Parameter	СС	GC + GG	p
Onset age, years	8.37 ± 4.95	8.6 ± 4.3	0.43

Morning stiffness, min	69.3 ± 73.6	61.2 ± 78.6	0.36

SJC	2.6 ± 3.4	5.2 ± 9.3	0.11

TJC	3.25 ± 3.5	6.35 ± 11.2	0.11

VAS, mm	29.95 ± 19.0	25.65 ± 16.1	0.21

RAI	6.15 ± 6.9	8.74 ± 12.5	0.2

DAS	2.22 ± 1.07	2.55 ± 1.6	0.21

DAS 28	2.89 ± 1.2	3.25 ± 1.3	0.17

Hemoglobin, g/l	129.4 ± 18.6	125.3 ± 19.1	0.24

Leukocytes, ×10^9^/l	7.7 ± 2.1	7.63 ± 3.01	0.45

Platelets, ×10^9^/l	261.0 ± 110.1	262.5 ± 93.6	0.48

ESR, mm/h	13.0 ± 13.8	13.22 ± 15.7	0.48

CRP, mg/l	6.6 ± 15.2	10.2 ± 21.2	0.26

Albumin, %	52.8 ± 8.9	52.0 ± 8.9	0.25

We did not detect significant differences in the frequency of patients with different genotypes treated with steroids. Twenty percent of children with the CC genotype and 27.7% of children with the G allele (CG + GG genotypes) received steroids (p = 0.44).

In regression analysis numerous markers of JIA activity influenced the presence of low bone mineral density for chronological age: morning stiffness (p = 0.01), SJC (p = 0.015), TJC (p = 0.01), VAS (p = 0.004), RAI (p = 0.04), DAS (p = 0.0006), DAS 28 (p = 0.01), platelet count (p = 0.007), CRP (p = 0.01) and albumin level (p = 0.03). Other factors (age of onset of JIA, duration of JIA, Hb, leukocyte count, and ESR) had no significant influence on the presence of low BMD. Girls with JIA who have the G allele had the combination of lower bone mineralization parameters with higher levels of osteocalcin, C-terminal telopeptides, ionized calcium, and total alkaline phosphatase (Tables 5, 6). A strong correlation between the G allele and bone mineralization parameters was found: BMC (r = -0.39), BMD (r = -0.31). A negative correlation with bone area (r = -0.4), due to lower anthropometry parameters, was found in carriers of the G allele. The frequency of low bone mineral density (LBMD) for chronological age differed among JIA subtypes: 11.8% in oligo JIA, 22.0% in poly JIA, and 54.8% in systemic JIA. Because the systemic JIA patients are those with the highest levels of inflammation and the most steroid use, the influence of the G allele is unlikely to be clinically significant in determining low bone mineral density.

**Table 5:** Markers of bone mineralization and metabolism in JIA girls with polymorphic GR genotypes.

Parameter	CC	GC	GG	**p**_**1**_	**p**_**2**_	**p**_**3**_
ВА, см^2^	51.9 ± 11.0	38.0 ± 12.6	32.3 ± 13.5	**0.00005**	0.11	**0.0001**

BMC, grams	43.5 ± 13.8	28.1 ± 16.8	19.6 ± 15.2	**0.0004**	0.07	**0.00007**

BMD, gms/см^2^	0.816 ± 0.15	0.677 ± 0.22	0.566 ± 0.19	**0.005**	0.06	**0.048**

BMD - Zscore (SD)	0.03 ± 0.85	0.007 ± 0.9	-0.931 ± 0.88	0.46	**0.003**	**0.002**

Osteocalcin, ng/ml	82.5 ± 5.1	87.3 ± 44.8	114.3 ± 39.8	0.37	**0.05**	**0.03**

CTТ, ng/ml	0.95 ± 0.34	1.02 ± 0.37	1.24 ± 0.42	0.28	0.09	**0.036**

PTH, pmol/ml	2.29 ± 1.23	1.9 ± 0.83	2.08 ± 0.64	0.11	0.25	0.26

Ca, mmol/l	2.4 ± 0.15	2.36 ± 0.14	2.38 ± 0.13	0.09	0.29	0.29

Ca^++^, mmol/l	1.02 ± 0.12	1.05 ± 0.11	1.1 ± 0.11	0.18	0.07	**0.017**

P inorganic, mmol/l	1.54 ± 0.2	1.61 ± 0.2	1.55 ± 0.2	0.1	0.23	0.44

TAP, U/l	293.5 ± 119.8	360.8 ± 116.1	328.8 ± 115.8	**0.01**	0.19	0.17

р_1 _- comparison between carriers СС and GC genotypes

р_2 _- comparison between carriers GC and GG genotypes

р_3 _- comparison between carriers СС and GG genotypes

**Table 6:** Markers of bone mineralization and metabolism in JIA boys with polymorphic GR genotypes.

Parameter	CC	GC + GG	p
BA, CM^2^	45.12 ± 16.1	45.26 ± 8.9	0.49

BMC, grams	32.79 ± 19.2	29.72 ± 13.1	0.32

BMD, gms/CM^2^	0.661 ± 0.21	0.642 ± 0.16	0.39

BMD - Zscore (SD)	-0.15 ± 1.06	-0.349 ± 1.33	0.34

Osteocalcin, ng/ml	130.5 ± 50.1	150.7 ± 54.5	0.21

CTT, ng/ml	1.46 ± 0.48	1.54 ± 0.45	0.36

PTH, pmol/ml	2.03 ± 0.9	2.75 ± 1.85	0.23

Ca, mmol/l	2.35 ± 0.16	2.38 ± 0.13	0.15

Ca^++^, mmol/l	1.12 ± 0.11	1.08 ± 0.09	0.11

P inorganic, mmol/l	1.6 ± 0.21	1.59 ± 0.16	0.43

TAP, U/l	376.0 ± 106.2	384.5 ± 127.3	0.41

## Discussion

Since their discovery in 1949, glucocorticoids have been commonly used for treatment of pediatric and adult rheumatic diseases. On one hand, glucocorticoids have strong and rapid anti-inflammatory effects; on the other hand, long-term use is associated with a many adverse effects and sometimes with eventual loss of therapeutic effect. Nearly 30% of adult rheumatoid arthritis patients have been reported to become glucocorticorticoid resistant (absence or failure of an expected response to treatment) in 3-6 months [7]. There are several mechanisms that may be important to resistance and susceptibility to glucocorticoids, including reduction of the number of glucocorticoid receptors and/or a reduced affinity for the ligand, polymorphisms in GR genes, and differences in other genes that modify the glucocorticoid response (transcription factors, post-translational modifications, and the activity of GRβ [7]).

In our study we have not detected an association between *Bcl*I genotypes and susceptibility to JIA, but we have shown an association of the G allele and GG genotype with both a younger age of JIA onset and markers of higher inflammatory activity and arthritis severity. We did not find any significant association of the GG genotype with subtype of JIA, but we did find an insignificant imbalance of subtypes between the two sexes (girl prevalence in polyarticular and systemic patients) and an insignificant GG genotype prevalence in these two JIA subtypes. These trends could be a reason that the detected associations occur mostly in girls, but perhaps a high prevalence of the GG genotype in polyarticular and systemic patients is related to the more severe course of these subtypes.

In previous reports, *Bcl*I polymorphic genotypes were not associated with a risk of rheumatoid arthritis in Caucasian patients, and there were no differences in genotype distribution in rheumatoid arthritis patients with and without erosions or the presence of the HLA-DRB1 allele [17]. Similar results have been reported in patients of Korean origin. In this study of adult RA patients, there was no association between *Bcl*I polymorphic genotypes and patients with rheumatoid factor, joint erosion and extra-articular complications [18]. In a recent study in a Greek RA population, the role of *Bcl*I and other polymorphisms in RA susceptibility was investigated. Among GRα intron 2 polymorphisms rs33388, rs33389, and *Bcl*I, and the GRβ variant rs6198, only the difference in rs33388 genotypes between RA patients and controls was marginally significant. However, several combined genotypes (haplotypes) of these polymorphic genotypes were related with RA susceptibility [9].

The association of *Bcl*I polymporphisms with other diseases besides RA has also been investigated, with varying results. In patients with multiple sclerosis, no increased frequency of *Bcl*I polymorphic genotypes and alleles was detected. There has been no evidence of peripheral glucocorticoid sensitivity in multiple sclerosis patients [19]. In childhood acute lymphoblastic leukemia, researchers have reported conflicting results. In one study no association between *Bcl*I genotypes and glucocorticoid susceptibility in vivo and in vitro was observed [20]. Another study showed an association between homozygosity for allele G of the *Bcl*I polymorphism and overall survival [21]. In the study of pediatric steroid-responsive nephrotic syndrome (SRNS) from Poland, no associations of polymorphic genotypes and alleles of the glucocorticoid receptor gene (*Bcl*I, rs33389 and rs33388) with nephrotic syndrome were observed. The distribution of individual polymorphisms and three-marker haplotypes was similar in healthy children and SRNS patients, but haplotype *Bcl*I G, rs33389 T, rs33388 A was associated with a higher glucocorticoid sensitivity [22]. In a population of Crohn Disease patients, a significantly higher frequency of the *Bcl*I polymorphism was found as compared to healthy volunteers [23].

The relationship between *Bcl*I polymorphisms and glucocorticoid sensitivity has been reviewed [24]. In healthy men, the GG genotype was associated with high cortisol levels compared with CC; healthy people with the GG genotype had enhanced sensitivity of skin, but low sensitivity of white blood cells, to glucocorticoids [25]. In an elderly Dutch population, the presence of the G allele was associated with lower cortisol levels after a dexamethasone suppression test, suggesting an increased sensitivity to GCs [13]. In cystic fibrosis patients, the GG genotype was associated with more severe lung damage (related to neutrophil infiltration of airways) and decreased glucocorticoid sensitivity [26]. In the Longitudinal Ageing Study Amsterdam in older men and women, females with the GG genotype had a higher cortisol level, and presence of the GG genotype was associated with lower trochanteric region bone mineral density in the total population [27]. Also, as previously noted, this polymorphism has been described to be associated with metabolic disturbances, such as increased blood pressure, increased abdominal obesity, and increased body mass index [28].

It should be emphasized that the way in which the *Bcl*I polymorphism may alter glucocorticoid effects is not known. As explained by Manenschijn et al., "This polymorphism is intronic, and its location does not involve a coding, regulatory or splicing part of the GR gene. It is possible that this polymorphism is in linkage with other variations, e.g., in the promoter region, or linked to other functionally important polymorphisms" [24].

In JIA, genetic factors can act differently in females vs males. For example, in another study, female JIA patients presented a higher frequency of a 14-bp insertion/deletion polymorphism as compared to healthy female children. This difference was observed in all JIA subtypes. No such difference was observed in RA patients [29]. In our study, the G allele and the GG genotype of the *Bcl*I polymorphism was associated with more active JIA only in girls.

## Conclusions

The present study has addressed the role of *Bcl*I GR gene polymorphism in JIA course, bone mineralization, and prognosis. In girls with JIA, the presence of the G allele was associated with an unfavorable arthritis course, a younger age of onset of arthritis, and some markers of higher inflammatory activity. We conclude that GR polymorphism could be a risk factor for more severe disease course or lower susceptibility to endogenous and exogenous glucocorticoids. Further investigation is required to test this hypothesis, which may provide new insights into JIA pathogenesis and the patient response to glucocorticoid treatment.

## List of abbreviations

JIA: juvenile idiopathic arthritis, RAI: Ritchie Articular Index, SJC: swollen joint count, TJC: tender joint count, VAS: visual analog scale, Hb: hemoglobin, L: leukocyte count, Pl: platelet count, ESR: erythrocyte sedimentation rate, CRP: C-reactive protein, DAS: disease activity score, DXA: dual-energy X-ray absorptiometry, BA: bone area, BMC: bone mineral content, BMD: bone mineral density, CTT: C-terminal telopeptides, PTH: parathyroid hormone, P: inorganic phosphate, TAP: total alkaline phosphatase, GR: glucocorticoid receptor, SRNS: steroid-responsive nephrotic syndrome

## Competing interests

The authors declare that they have no competing interests.

## Authors' contributions

MMK acquired the data. AAK and MVM carried out the molecular genetic studies. MMK did the literature search and the statistical analysis, and wrote the paper. LAS and VIL participated in study design and coordination and helped to draft the manuscript. MMK, LAS and VIL interpreted the data and were responsible for the manuscript preparation. All authors read and approved the final manuscript.
